# Characterization of Roman and Arabic Mural Paintings of the Archaeological Site of Cercadilla (Cordoba, Spain)

**DOI:** 10.1155/2019/3578083

**Published:** 2019-07-28

**Authors:** Andrea Gil-Torrano, Auxiliadora Gómez-Morón, José María Martín, Rocío Ortiz, Mª del Camino Fuertes Santos, Pilar Ortiz

**Affiliations:** ^1^Universidad Pablo de Olavide, Department of Physical, Chemical and Natural Systems, Carretera de Utrera Km 1, ES-41013 Sevilla, Spain; ^2^Andalusian Historical Heritage Institute (Seville, Spain), Avenida de los Descubrimientos S/N, 41092 Sevilla, Spain; ^3^Andalusian Agency of Cultural Institutions, Andalusian Council of Culture, Seville, Spain

## Abstract

The archaeological site of Cercadilla (Cordoba, Spain) includes a complete chronological sequence from the 3rd to 12th centuries. The most relevant monument is a Roman palace dated between the end of the 3rd century and the beginning of the 4th century AD. It is believed that it was the headquarters of the Emperor Maximiano Herculeo. A bathtub with mural paintings has been found in the thermal zone of the palace. Regarding the occupation of the archaeological site in the medieval period, it should be pointed out that two houses with mural paintings were found; these belong to the Caliphal era (10th-11th centuries). During the Caliphal era, the archaeological site was mostly occupied by one of the large suburbs surrounding the walled city. Cercadilla was gradually abandoned; this process starts at the beginning of the 11th century. This study is focused on the analysis of pigments and preparatory layers of red and white mural paintings of the Roman period in the bath zone and on the analysis of pigments in mural paintings in two houses of the Caliphal era. In the thermal zone, the walls have a white mural painting with vertical and horizontal red bands, while the walls in the two Caliphal houses present the red mural painting decorated with white stripes. Techniques such as Optical Microscopy (OM), Scanning Electron Microscopy in combination with Energy Dispersive X-ray Microanalysis (SEM-EDX), X-ray Diffraction (XRD), micro X-ray Diffraction (*μ*-XRD), Wavelength Dispersive X-ray Fluorescence (WD-XRF), and Fourier Transform-Infrared Spectroscopy (FT-IR) have been used to study the mural paintings of this archaeological site. The results allowed to determine the composition of the materials used and to understand the differences between the technologies employed in Roman and Caliphal remains studied.

## 1. Introduction

**Figure 1:**
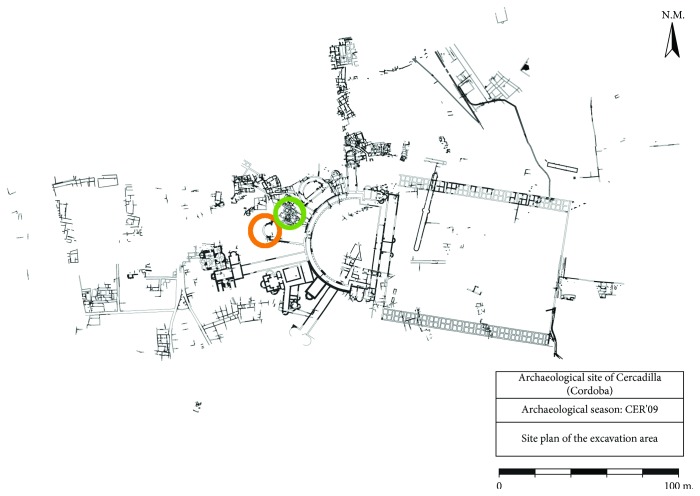
Archaeological site of Cercadilla [3], where the location of Roman and Arabic mural paintings is circled in green and orange, respectively.

The archaeological site of Cercadilla, located in Cordoba (Spain), is an important archaeological site (Figure 1). It was occupied from the 1st century BC to the post-Caliphal era, and it reached its peak with the construction of a large palace complex of the tetrarchy era (293-304 AD) [1, 2].

Through numerous studies in the site of Cercadilla, it has been deduced that the palace complex was built between the late 3rd century AD and the early 4th century AD (in particular, between 293 and 305 AD) and that it rose above an early imperial suburban Roman villa. Besides, it has been considered that the complex was the headquarters of the Emperor Maximiano Herculeo. A wide historical sequence is encompassed during the occupation of the archaeological site during the medieval period, with the following three major historical phases: (1) a late antiquity period (6th-8th centuries), in which a part of the ancient Roman palace was used as a Christian worship center; (2) an Emiral phase that comprised three periods (8th-10th centuries); (3) a Caliphal era (10th-11th centuries), in which the archaeological site was mostly occupied by one of the large suburbs that surrounded the walled city. From the Caliphal era have been documented more than sixty houses, public buildings, a souk, a possible *funduq* and baths, and religious buildings (including a mosque in the basement of the today's bus station). The gradual abandonment of the Cercadilla quarters began on the beginning of the 11th century [3].

The archaeological site covers about two hectares. However, a great part is hidden under the urban planning; its size is believed to be larger. Besides, presently, the site is devastated and covered by the construction of the railway station of the city. Despite the partial destruction, the archaeological site is one of the most important heritage values in the city [4].

The palace complex built during the late Roman period comprises a closed building oriented towards the northwest of the walled city. It is arranged in two different construction bodies: one of them with a military role as a host and the other one with a strictly palatine character [5].

The palace is organized around a large cryptoporticus in exedra with a portico disposed over it, around which the different rooms that form part of the ensemble are distributed radially [2]. The main building of the whole archaeological ensemble is a big reception aula with a basilical ground plan, which is located in the center of the palace. The baths were built in the north of the basilical aula, whose small size allows to deduce that they were conceived to a private character [5].

Regarding the Caliphal era, it is important to point out that two Caliphal houses were found in good state of conservation when the top of the basilical area of the palatine complex was excavated. Both houses maintained the preparatory layer with almagre and the decoration with white stripes when they were excavated [4]. Conservation treatments were performed in 2005 after two years of being exposed to weathering agents, because the mural paintings showed a high disintegration, general weathering of pigment layers, and lack of pigment adherence. A consolidation treatment was carried out on some intern areas of the walls. In addition, the calcareous concretions that covered all the wall were removed by mechanical methods. Finally, a protection film of ethyl silicate was applied; the cracks and crevices were filled as well as the edges of the mural paintings with mortar of lime and sand in order to prevent the water seepage.

In the case of the thermal zone of the Roman palace, the main construction technique used was *opus caementicium* with *opus signinum* coating. The *opus caementicium* is achieved through the amalgamation of lime and sand with stone remains. The *opus signinum* technique was employed in the Roman period to provide the materials with an impermeable character, characterized by the use of a lime mortar, sand, and fragments of pottery vessels or bricks [6]. The mural paintings were in good state of conservation when they were discovered, but they present crust and concretions. The conservation process performed in this zone consisted of a cleaning treatment, because the surface of the walls was covered by insoluble salts (salt crusts and concretions) as well as soluble salts. It was decided to maintain the calcareous concretions, and the thermal zone was protected with geotextile.

Preliminary studies performed on Cercadilla mural paintings [7, 8] focused this research on the comparison between the paintings of the thermal bath which still conserves a white mural painting with red vertical stripes and red decorations on the edges and the mural paintings found in the two houses of the Caliphal era. The ornamentation of Arabic buildings used to be dichromatic (commonly red and white, which is also the case of this study) and with geometric and repetitive designs [9]. It is generally stated that external wall paintings are specially affected by changes in temperature and humidity, deteriorating phenomena associated with soluble salts, and microbiological activity, among others [10]. There are a variety of methods that can be used to investigate ancient pigments and mural paintings; the most suitable methods for each study are chosen depending on the type and amount of sample available [11]. Several complementary analytical techniques are usually required to provide insight into the degradation processes of the materials in the wall layers, as well as to gain understanding on the composition of these materials and the painting techniques used [12]. In this study, a range of analysis was performed on samples taken from mural paintings to investigate the current state of conservation of the archaeological site. These include colorimetry and macrophotography, Optical Microscopy (OM), Scanning Electron Microscopy in combination with Energy Dispersive X-ray Microanalysis (SEM-EDX), X-ray Diffraction (XRD), micro X-ray Diffraction (*μ*-XRD), Wavelength Dispersive X-ray Fluorescence (WD-XRF), and Fourier Transform-Infrared Spectroscopy (FT-IR).

Each one of the building complexes is unique, and the study of the materials found in them can give relevant information about the civilizations that inhabited the region in these periods of history. In this work, a complete multiapproach characterization of mural paintings from the Cercadilla archaeological site is presented, for the first time, bringing valuable information about the state of conservation of the site as well as the materials and techniques used by Roman and Arabic civilizations.

## 2. Materials and Methods

**Figure 2:**
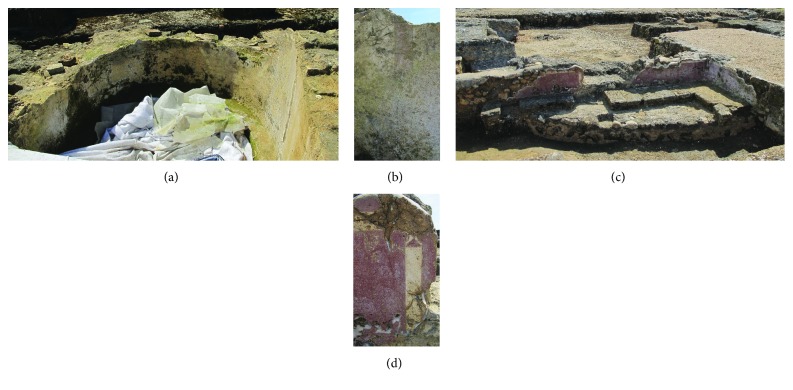
Areas with mural painting remains: (a) the bath with Roman mural painting is showed; (b) a detail of one of the red stripes; (c) one of the Arabic houses are showed; (d) a detail of Arabic mural painting.

Nineteen samples of Roman and Arabic mural paintings were taken from the Cercadilla archaeological site (Table 1). The Roman samples belonged to the thermal zone of the palace; they were located in one of the three bathtubs that define a room destined to a cold water bath (*frigidarium*). Specifically, the paintings were found between two rectangular baths, in a central semicircular bath. On the other hand, the Arabic samples were found in two houses attributed to the Caliphal suburban, named as zone 1 (Is-01, Is-02, Is-03, Is-04, Is-05, and Is-06) and zone 2 (Is-07 and Is-08). The samples were taken from not-treated zones. The mural paintings were white and red in both Roman and Arabic samples. In the case of the Roman ones, the paintings had vertical red stripes and red horizontal paint on the edges, as decoration motifs over the white paint. On the other hand, the Arabic paintings presented red decoration with vertical and horizontal white stripes on the edges and vegetal motifs in one of the houses (Figure 2).

The study of the mural paintings was carried out through several techniques including *in situ* techniques such as colorimetry (using a portable Colorimeter X-Rite Series SP60 to obtain the *L*^∗^*a*^∗^*b*^∗^ attributes) and macrophotography (using a Zarbeco MiScope 2000 digital microscope with visible, infrared, and ultraviolet light), in order to observe in detail the paintings and to document their state of conservation. A large amount of points were taken with the colorimeter to ensure that a representative group of measurements were gathered. Macrophotographs were also taken in different areas of the Roman and Arabic mural paintings.

The cross section of the painting (stratigraphy) was studied with Optical Microscopy (OM) (Leica DM4000M model), which allowed to determine the number and sequence of layers as well as their thickness. The analysis of the cross section of the samples can provide a number of important data on the technique used in its preparation and its state of conservation. The analysis of the samples with an optical microscope allows to appreciate the stratigraphic structure, the colours of the different layers with pigments and the support, the textural aspects, thickness in the samples, and also the presence of inclusions with different sizes of particles [13]. The microscopic study included observation with UV light and staining-based techniques to investigate organic binders on cross sections. The staining tests were acid fuchsin for identifying animal glues, gelatines, and egg proteins; Sudan black B, for lipids; and Lugol solution, for starches.

In order to determine the elemental chemical composition of the different layers (pigment and preparatory layers), the pigments used were identified using Scanning Electron Microscopy in combination with Energy Dispersive X-ray spectrometry (SEM-EDX) (JEOL Jsm-5600LV with Oxford microanalyser INCA Energy 200). Electron microscopy coupled with energy dispersive X-ray spectrometry is now one of the most significant and powerful analytical instruments that are often used for the characterization of historical pigments [12]. The test with microscope and chemical microanalysis allows to discover the stratigraphic structure and composition of the materials that define the sample [14].

Eight of the samples were prepared for OM and SEM-EDX, four of which were Arabic (Is-06 and Is-08 as red samples, Is-02 and Is-03 as white samples), and the other four were Roman (Rom-01 and Rom-02 as red samples, Rom-03 and Rom-06 as white samples). The samples were included in a bicomponent methacrylate resin of self-curing and prepared in cross section. Four samples (Is-06, Is-03, Rom-02, and Rom-03) were coated with a thin layer of gold to improve conductivity and to avoid charging during the SEM-EDX examination, using a sputter coater Scancoat Six, Pirani 501 Model.

X-ray Diffraction (XRD) analysis was carried out in a Bruker D8 Advance diffractometer to analyse the mineralogical composition of mortars. The Rietveld refining method has been used in the quantification by XRD applying the Match! software version 3; the results were normalised. Two mortar samples were analysed in total (one Arabic (Is-08) and one Roman (Rom-10) mortars). Both samples were grinded to powder and then sieved to achieve a suitable grain size [15].

Moreover, micro X-ray Diffraction (*μ*-XRD) (Bruker Discover X-ray diffractometer) was carried out to determine the mineralogical composition of the mural paintings. The analyses were performed nondestructively on the surface of the painting samples. This technique is very sensitive and recommended for painting layers in order to detect the presence of small grains of pigments. For this analysis, four samples were selected: two Roman and two Arabic painting samples (Rom-05 and Rom-09; Is-05 and Is-03, with red and white paintings, respectively).

Arabic and Roman mortar samples (Is-08 and Rom-10) were also examined by Wavelength Dispersive X-ray Fluorescence (WD-XRF), with a PANalytical XRF equipment AXIOS Model. Powder pellets were prepared to carried out the analyses by WD-XRF: 1 g of grinded powder mortar was mixed with lithium-tetraborate (66 wt%) and lithium metaborate (34 wt%). The mixture was processed for loss on ignition (LOI) at 1050°C using the Eagon 2 fusion instrument (PANalytical).

Finally, Fourier Transform-Infrared Spectroscopy (FT-IR) was carried out to analyse mortars and painting samples (Rom-02, Rom-03, Is-02, Is-03, Is-06, and Is-08) using a PerkinElmer Spectrum Two with a diamond crystal (Attenuated Total Reflectance (ATR)). The samples were pressed against the ATR crystal nondestructively without grinding. The spectra were collected in the range 450-4000 cm^−1^ employing 16 scans average for each spectrum and a spectral resolution of 1 cm^−1^. The references employed for the FT-IR wavenumbers are published in the Infrared and Raman Users Group Spectral Database [16] and in the Spectral Database for Organic Compounds (SDBS) [17].

## 3. Results and Discussion

### 3.1. Colour and Surface Description

Macrophotographs and colorimetry are important to establish a data record to aid future restorations. These methods allow to judge whether the conservation treatments of painted surfaces have altered the appearance of the paintings [18].

**Figure 3:**
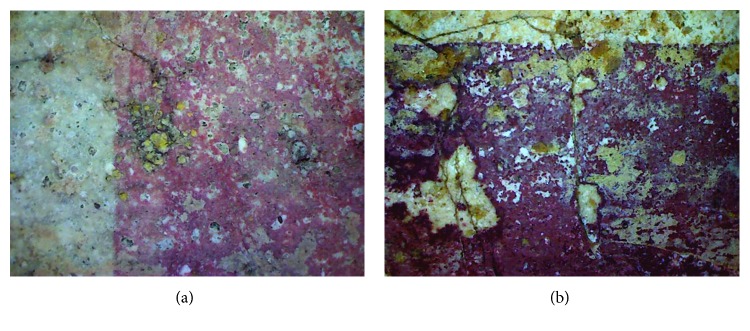
Macrophotographs: (a) Roman mural painting; (b) Arabic mural painting.

From the photographs taken (both general and macro), the Roman mural painting seems to be cohesive but with clear signs of deterioration on the surface of the pictorial layer (Figure 3(a)). Many alterations have been detected such as the presence of white salts on the painting surface and lichens (yellow, green, and brown) and the presence of insects and others animals. In addition, some mechanical damages were observed in the form of cracks. The superficial crust goes through several colour tones, from a whitish, another cream-coloured, to darkish tones, covering practically all the bath surface. As the presence of red paint was uncertain, some surfaces were measured with the colorimeter. The *L*^∗^*a*^∗^*b*^∗^ attributes obtained confirmed that these areas contained red paint, reinforcing the previous supposition and concurring with the spatial arrangement of the paintings.

The Arabic mural painting looks less cohesive and powdered than the Roman ones; hence, it presents various marks of flaking (detachment of the paint layer), even though it has been previously restored. Moss and lichen colonization penetrates into the inner layers and may produce fractures and loss of material. The insects observed over the wall might contribute to this mechanical damage due to their dug galleries. One of the macrophotographs taken is shown in Figure 3(b), where cracks and fissures, as well as loss of the paint layer and efflorescences, can be observed.

As expected, the colorimetric data showed a clear separation between the white paint group and the red paint ones. In the case of the zones with white mural painting, the *a*^∗^ attribute (red-green values) had a positive value higher than what is expected. This is possibly due to the salt superficial layer which covers almost all the painting surface with a brownish tone.

### 3.2. Stratigraphy of the Paintings

**Figure 4:**
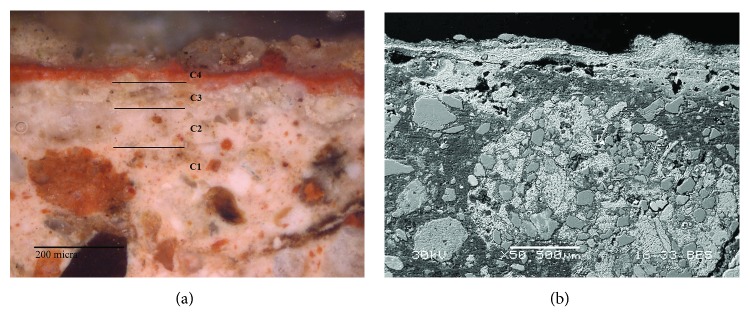
(a) Microphotograph carried out by Optical Microscopy of the Roman red mural painting sample Rom-01, where the pictorial and preparatory layers are shown. (b) Backscattered electron image of the Roman red mural painting sample (Rom-01). Four layers were observed in the Roman samples (C1-C4), which present a different grain size from the inner (coarser) to the top (thinner).

At least four layers (Figure 4(a)) with different grain size particles were revealed by OM in the Roman red mural painting samples (Rom-01, Rom-02, Rom-04, and Rom-11). In the deepest layer (named as C1), the preparatory layer is observed. This is composed of coarse grains with a range of grain sizes comprising different minerals, even fossil fragments. This deepest layer was applied to equalize the mural substratum. Besides, this layer also contained ceramic and brick fragments. This goes in line with the *opus signinum* technique, which was commonly applied to keep the humidity in the bath stable. Over this first layer, there are two more preparatory layers, named in Figure 4 as C2 and C3 layers. The thickness measurement in C2 layer ranges from 100 to 120 *μ*m whereas in C3 layer, range is from 45 to 50 *μ*m. The grain size in these layers is smaller than in layer C1 where some grains even reach 200 *μ*m. The aim of the two layers was to prepare the surface to apply the paint layer in a uniform way. The outermost layer corresponds to the pictorial layer, named C4. The thickness of this layer ranges from 10 to 20 *μ*m. An irregular brown coating is observed covering this last layer which is attributed to dirt (i.e., salts and lichen colonization present on the mural surface).

**Figure 5:**
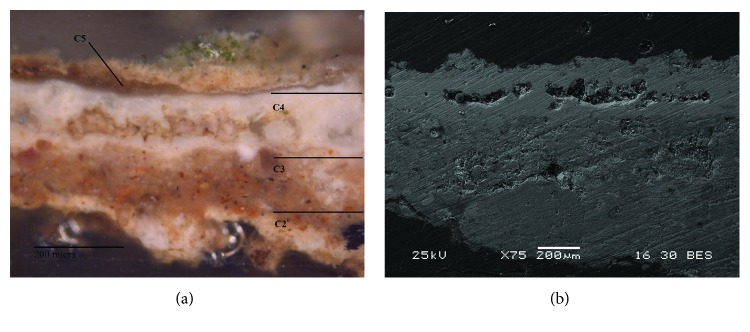
(a) Microphotograph carried out by Optical Microscopy of the Roman white mural painting sample Rom-06, where the pictorial and preparatory layers are shown. (b) Backscattered electron image of a Roman white mural painting sample (Rom-03). Two preparatory layers (C2 and C3) and the pictorial layer (C4) are shown. In addition, an efflorescence that cover the pictorial layer is shown (C5 layer).

White painting samples of the Roman period (Rom-03, Rom-06, and Rom-07) showed at least five layers (Figure 5(a)). The first three layers (C1, C2, and C3) correspond to the preparatory layers, where thickness ranges from 100 to 120 *μ*m in the case of C3 layer. Similar to the red painting samples, the grain size of the white paint also decreases with the proximity to the pictorial surface. The paint layer was named C4, with thickness from 120 to 150 *μ*m. Above it, there was another layer (C5) that possibly was an efflorescence that had emerged to the surface, covering part of all the white paint providing it with a darker tone, where thickness ranges from 5 to 30 *μ*m. Finally, dirt and green vegetation were present over the C5 layer. The neat separation between the pigment and the last preparatory layer further suggests that the latter was already dried when it was decorated [19–24].

Roman samples (Rom-01 and Rom-06) revealed red fluorescence to ultraviolet light, which was attributed to the presence of ceramic fragments added to increase the impermeability of the structure. Moreover, some grains of these samples showed blue fluorescence, but it may be due to the presence of impurities.

The application of multiple layers with an increasingly fine granulometry is common in the Roman technique while in the case of the Arabic mural painting, the number of layers is smaller.

**Figure 6:**
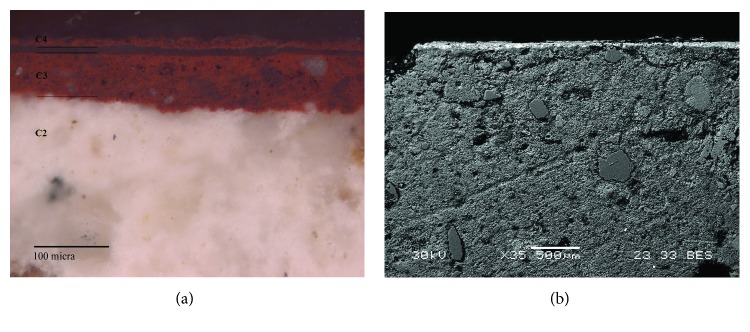
(a) Microphotograph carried out by Optical Microscopy with 20 magnification of the Arabic red mural painting sample Is-08, where the pictorial layers and one preparatory layer are shown. (b) Backscattered electron image of the Arabic red mural painting sample (Is-08). A detail of the two overlapped painting layers (C3 and C4) was observed.

The Arabic mural paintings showed at least four layers (Figure 6(a)). Red mural painting samples (Is-06, Is-07, and Is-08) were composed of two preparatory layers and two red painting layers. With regard to the two preparatory layers, the deepest layer (C1) present thicker grains than the overlapped preparatory layer (C2), in order to prepare the surface to apply the paint layer.

Another point was the whitish tones present in the Arabic preparatory layers, probably because the Arabic samples do not present remains of ceramic and brick fragments, like in the case of Roman samples which are hydraulic, and that is why its consistency is powdery and loosely cohesive. Looking in the red pictorial layers again, the presence of two layers with the same colour overlapping itself catches the attention. The deepest, named C3, is wider than the layer immediately above, where thickness ranges from 50 to 80 *μ*m, whereas C4 thickness layer goes from 10 to 15 *μ*m. The presence of a dirt layer between the two red layers with a thickness of 5-10 *μ*m should be pointed out. These suggest that initially the mural painting only had one pictorial layer and the wall was repainted later over the first layer, capturing the dirt between them.

**Figure 7:**
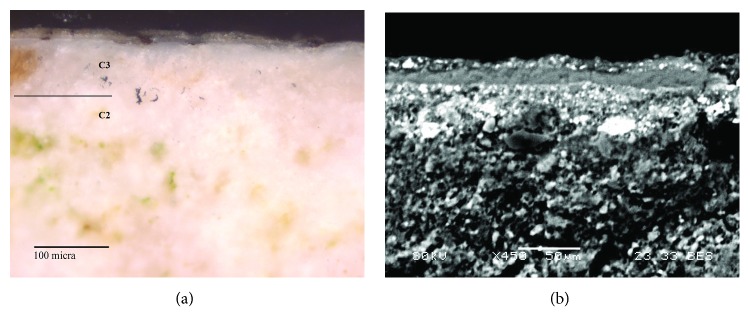
(a) Microphotograph carried out by Optical Microscopy with 20 magnification of the Arabic white mural painting sample Is-02, where the pictorial layer and one preparatory layer are shown. (b) Backscattered electron image of the same sample (Is-02). The white pictorial layer is shown in detail, where the heterogeneity of the pigment grains is observed.

The white Arabic paint (Figure 7(a)) (Is-01, Is-02, Is-03, and Is-04) presented at least three layers. The deepest layers (C1 and C2) are preparatory layers, changing from coarser grains to finer grains as they rise to the pictorial surface. The last layer (C3) belongs to the white pigment, which is thinner (from 50 to 70 *μ*m), while another overlapped dirt layer can be observed. In this case, the lichen colonization goes through the preparatory layers damaging the deeper layers. In addition, some black inclusions as well as vegetal remains were observed in the preparatory layers, likely added during the manufacturing process.

In the manner of Roman paintings, the Arabic paintings show also a clear distinction between the preparatory layers and the colour layer, which is a characteristic of the *secco* technique [19–24]. However, the lower number of preparatory layers in the case of Arabic wall paintings compared to the high number of layers of plaster applied in Roman samples is worth highlighting [25, 26].

In addition, a binder based on proteins was detected in the staining-based analyses performed to determine the organic binders of the *secco* technique. Arabic and Roman samples (Is-03, Is-06, Rom-02, and Rom-03) showed a positive response to the acid fuchsin staining test and negative responses for the other staining tests such as Sudan black B and Lugol solution. FT-IR analyses did not allow determining the proteins employed.

### 3.3. Chemical and Mineralogical Characterization

**Figure 8:**
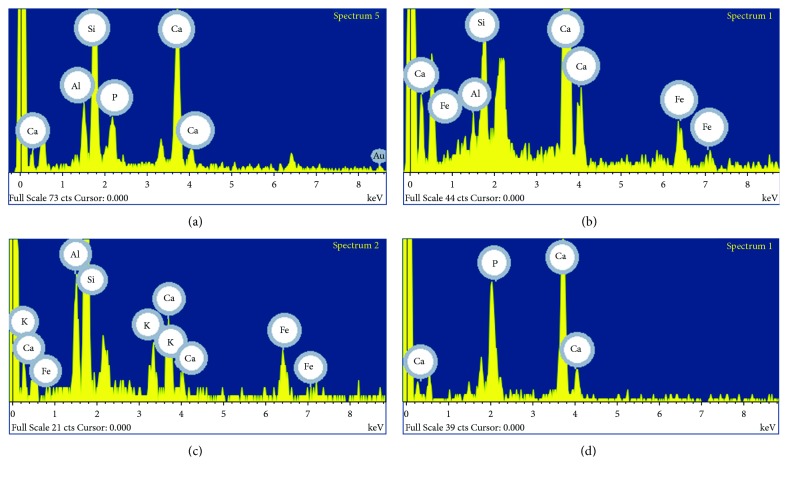
Spectra obtained by SEM-EDX in the Roman samples Rom-02 (red) and Rom-03 (white). (a) Calcium, aluminium, silicon, and phosphorous were detected in an area of the preparatory layer (Rom-02). (b) Calcium, silicon, aluminium, and iron were detected in point analysis of the pictorial layer (Rom-02). (c) Calcium, silicon, aluminium, potassium, and iron were detected in an area of the preparatory layer (Rom-03). (d) Calcium and phosphorous were detected in point analysis of the pictorial layer (Rom-03).

**Figure 9:**
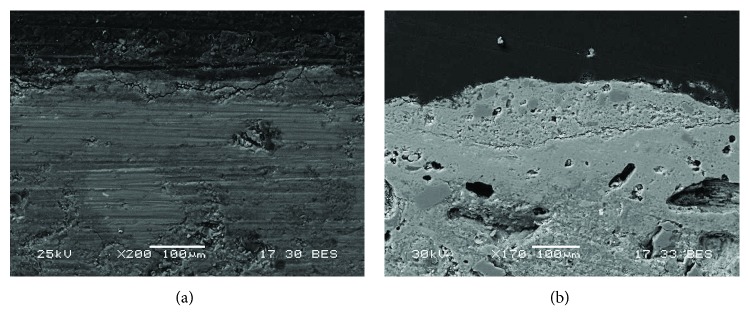
Backscattered electron images of two Roman samples. A detail of the painting layer is shown: (a) Rom-02 that presents red pigment, where the homogeneity of the grain is observed; (b) Rom-03 that corresponds to a white mural painting sample, which presents a high heterogeneity of grain size.

**Figure 10:**
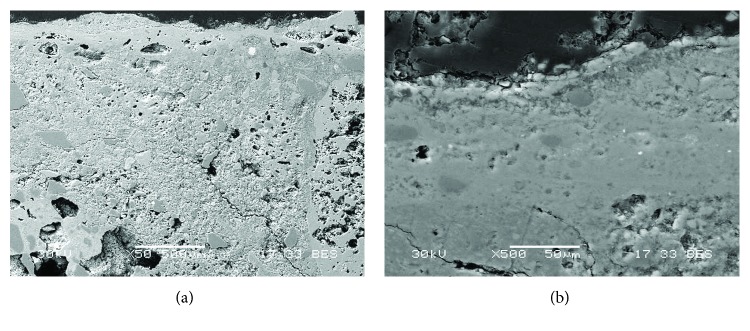
(a, b) Backscattered electron images of the Rom-03 sample which presents white pigment. (b) A detail of the pigment layer is shown. In addition, some mechanical damages as cracks were observed.

**Figure 11:**
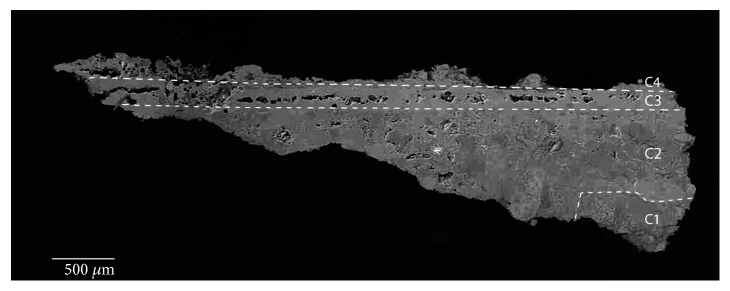
Backscattered electron image of the Roman red mural painting sample (Rom-02), where the preparatory layers (C1-C3) and the pictorial layer (C4) are shown.

**Figure 12:**
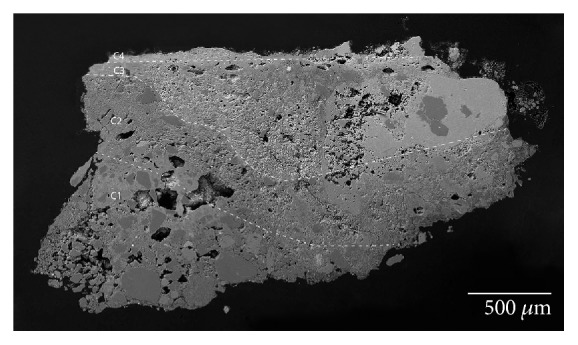
Backscattered electron image of the Roman white mural painting sample (Rom-03), where the preparatory layers (C1-C3) and pictorial layer (C4) are shown.

Preparatory layers of the Roman mural painting showed a high amount of calcium and, to a lesser extent, silicon and aluminium (Figure 8) by SEM-EDX analyses. Calcium, silicon, and aluminium were detected in red samples (Rom-01 and Rom-02) and white samples (Rom-03 and Rom-06) (Figures 4(b), 5(b), and 9–12). These results suggest that the preparatory layer was mainly composed of calcite (CaCO_3_) (Table 2).

The presence of calcium carbonate (calcite (CaCO_3_)) in high amounts in the preparatory layers is explained by the use of slaked lime (calcium hydroxide (Ca(OH)_2_)) as a raw material, which undergoes a process of carbonation by reaction with atmospheric CO_2_. Another reason to the presence of calcite in the preparatory layers could be due to the addition of sand and gravel CaCO_3_ as an inert material [25]. Calcite (CaCO_3_) has been used since antiquity, especially in preparatory layers and to a lesser degree as a pigment. It can be natural or artificial in origin. Its natural varieties include chalk, which is a white, soft, and porous rock, composed of an accumulation of the remains of marine microorganisms [27]. In this case, several fossil remains were observed in the inner layers of the Roman mural paintings, confirming its natural origin.

Silica and albite were in low proportion (Table 3) in the mortar lime, likely added as sand during the manufacturing process.

Besides, phosphorus was detected in the preparatory layers in two Roman samples (Rom-02 and Rom-03), indicating the presence of crushed bones (Ca_3_(PO_4_)_2_) (Figures 8(a) and 8(d)). The preparatory layers showed also the presence of iron, silicon, aluminium, and potassium (Figure 8(c)). The presence of these elements indicates that ceramic fragments were added in the mortar manufacturing; this goes in line with the red tone observed in the preparatory layers of the Roman paintings. Calcite was used as a white pigment in the Roman paintings.

Furthermore, the red layers present calcium, iron, aluminium, and silicon (Figure 8(b), Table 2); therefore, it is concluded that red earth was employed as red pigment [28]. Moreover, the preparatory layer was compound of a mixture of slaked lime and aggregates such as sand or ceramic materials in the Roman period. It was improved by adding the silica and alumina contained in volcanic ash, known as pozzolanic materials [29]. This fact explained the presence of those elements in the samples. The use of those materials was common, particularly in the preparation of mortars and plasters used in damp areas to improve the mechanical strength of these buildings [20].

Regarding the XRD analysis, the mineral phases identified in the Roman mortar sample (Rom-10) were mostly calcite (CaCO_3_) and quartz (SiO_2_) and, in a less extent, phyllosilicate minerals (Table 3).

The high calcium content observed suggests that it is the main component of the Roman mural painting preparatory layers, which corroborate the results obtained by SEM-EDX. The elevated silicate mineral content in this sample (Rom-10) could confer impermeable properties to the baths (*opus signinum* constructive technique).

These kinds of mortars are more resistant in the outdoors due to the possibility of a pozzolanic reaction between the ceramic aggregates and lime. That reaction can be contributing to the better behaviour (more internal cohesion and durability) of these mortars [30]. The preparatory layers of the Roman samples (Rom-01, Rom-02, Rom-03, Rom-04, Rom-06, Rom-07, and Rom-11) had a reddish tone due to the presence of ceramic fragments according to OM observations.

Moreover, gypsum was detected on the surface of Roman mural paintings (Rom-05 and Rom-09) by *μ*-XRD analysis as a result of alteration of inner layers (Table 4).

Chemical composition of mortars analysed by WD-XRF is shown in Table 5. Silicon, aluminium, and calcium were observed as the main elements in the Roman mortar (Rom-01). The presence of other elements such as iron and magnesium was also identified in lower quantities. Remarkably, the amount of silicon was significantly superior to the amount of calcium. This fact could be attributed to impurities in the original limestone, or most probably, it could indicate that quartz was added to avoid shrinking and crack formation in the paint [31]. Likewise, aluminium was found in a slightly higher concentration than calcium. These results could be associated with the addition of several ceramic fragments during the mortar manufacture in order to confer a greater resistance and impermeable properties. Moreover, a slight quantity of phosphorus was observed. This element was related to the possibility of crushed bone addition already mentioned in the SEM-EDX analysis.

**Figure 13:**
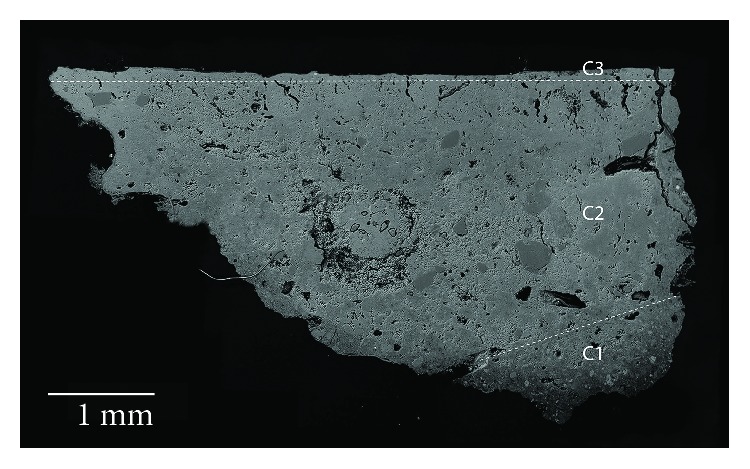
Backscattered electron image of the Arabic white mural painting sample (Is-03), where the preparatory layers (C1-C2) and pictorial layer (C3) are shown.

**Figure 14:**
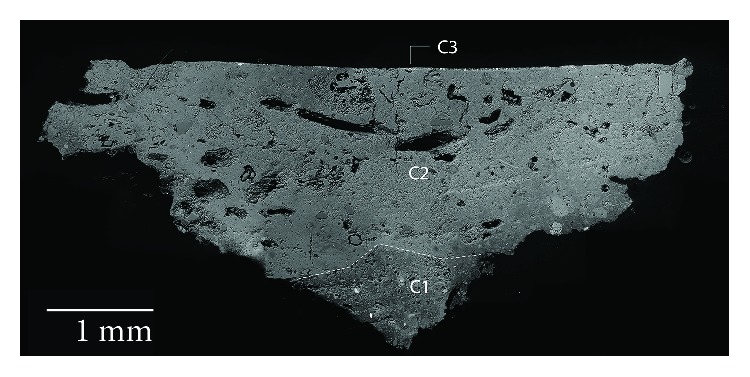
Backscattered electron image of the Arabic red mural painting sample (Is-06), where the preparatory layers (C1-C2) and pictorial layer (C3) are shown.

**Figure 15:**
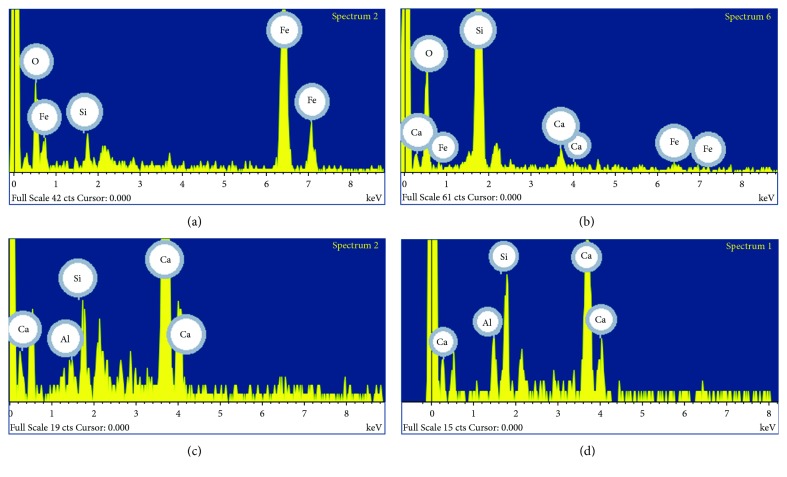
Spectra obtained by SEM-EDX in the Arabic samples Is-06 (red) and Is-03 (white). (a) Iron and silicon were detected in point analysis of the red pictorial layer (Is-06). (b) Silicon, calcium, and iron were detected in point analysis of the red pictorial layer (Is-06). (c) Calcium, aluminium, and silicon were detected in an area of the preparatory layer (Is-06). (d) Calcium, aluminium, and silicon were detected in the white pictorial layer (Is-03).

According to SEM-EDX results, calcium is the most abundant element in the Arabic mural painting (Is-02, Is-03, Is-06, and Is-08) and silicon is the second element in proportion (Figures 13 and 14). Both elements were detected in the analysis of the preparatory layers (Figure 15(c), Table 2). The deepest layer of preparation had more quantity of quartz grains, and they were larger than the upper layers where the quartz grain are smaller and in less quantity to obtain a homogeneous surface to apply the paint layer. The Arabic mural painting manufacture is different from the Roman ones in the number of preparatory layers, only two layers while Roman employed up to three layers of mortars with differing quantities of quartz. A similar technology process has been found in other Arabic mural paintings [22, 23, 25]. Sulphur was not detected in area analysis in the preparatory layer of the Arabic samples, whereas some small grains in the preparatory layer that contained sulphur and calcium were observed. This suggest that gypsum was present in the preparatory layer in a very low proportion.

**Figure 16:**
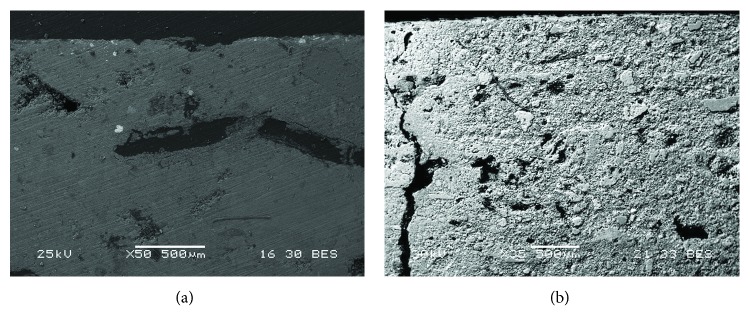
Backscattered electron images of two Arabic samples. (a) Is-06 presents red pigment. In addition, vegetal remains were observed, as well as a great homogeneity of the grains of the preparatory layer. (b) Is-02 corresponds to a white mural painting sample. Mechanical damages (cracks) and the heterogeneity of the grains were observed in the preparatory layers.

**Figure 17:**
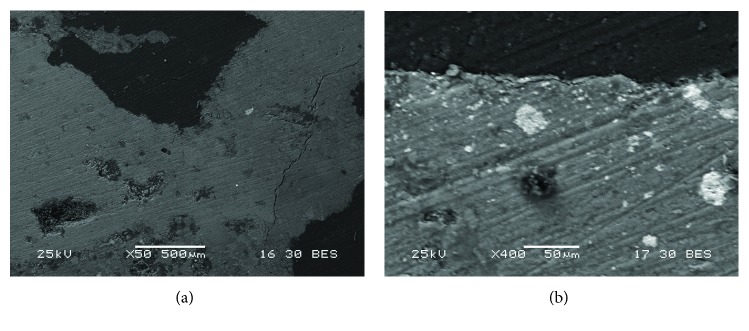
Backscattered electron images of an Arabic red mural painting sample (Is-06). (a) A crack present in the preparatory layer is shown. (b) A detail of the painting layer is observed.

Several pigment grains with a high amount of iron (33-57%) were observed by SEM-EDX (Figures 6(b), 14, 15(a), 16 and 17) in red Arabic samples (Is-06 and Is-08). Silicon, aluminium, and iron were also detected in other grains with less frequency in the red pictorial layer (Figure 15(b)). This result suggests that red ochre pigment was employed, a mixture of hematite (Fe_2_O_3_) and red earth. Hematite was commonly employed in Andalusia as a pigment because it was easy to prepare or obtain and because of its low cost [9].

The Arabic white samples (Is-02 and Is-03) showed similar results in the preparatory layers (Figures 7(b), 13, and 16). That means that preparatory and pigment layers are composed of calcite (Table 2). In fact, the white pigment used was calcite, although with a thinner grain than in the case of the preparatory layers.

The mineralogical phases found in the Arabic mortar sample (Is-08) by XRD analysis have been identified as calcite, as a major component, and quartz and gypsum as minor components. In addition, a small amount of albite, a sodium aluminosilicate, was detected (Table 3).

Calcite as a major compound of the preparatory layers was observed in the Arabic painting samples (Is-03 and Is-05) by *μ*-XRD analysis (Table 4). In minority amounts, silica was obtained, like in the case of the XRD analysis; likewise, quartz was also found in the SEM-EDX analysis as small grains, which could be added intentionally during the manufacturing of the mural painting or due to the presence of this mineral in the raw materials as an impurity, as it was already supposed in the prior techniques. Regarding the red painting layers of the Arabic samples, hematite was identified as main pigment.

These results reveal some compositional differences between Arabic and Roman samples. Gypsum was present in Arabic samples (Is-08) as a component in mortars (Table 3), while it was not in mortars of the Roman samples (Rom-10). On the other hand, phyllosilicate minerals were only detected in Roman mortars, likely due to the type of sample (bath zone) where the hydraulic nature was more necessary. Albite appears only in Arabic mortars in small quantities. These additives (phyllosilicates) modify the porosity, the volume, and the contraction of the mortars, providing mortars with this characteristic hydraulic nature [32].

The presence of gypsum on the surface of Roman and Arabic painting samples (Rom-05, Rom-09 and Is-03, Is-05, respectively) detected by *μ*-XRD can lead to suppose that it could be associated with alteration products from the inner layers (Table 4).

Besides, according to the prior results, calcium was found as a major component of the Arabic mortar (Is-08 sample) in the analyses performed by WD-XRF (Table 5), followed by a low percentage of silicon and, in minor quantities, other elements (aluminium, iron, and magnesium). That silicon content could be due to the addition of quartz during the manufacture. Besides, the low content of aluminium, iron, and magnesium might be associated with phyllosilicates; similar results have been observed in other Arabic mortar samples of the same periods [22, 23, 25].

Finally, the mural painting samples have been characterized by FT-IR (Rom-02 and Rom-03; Is-02, Is-03, Is-06, and Is-08). Regarding the mortars of the Arabic samples (Is-02, Is-03, Is-06, and Is-08), carbonates with very intense peaks at 1404 cm^−1^, 873 cm^−1^, and 712 cm^−1^, owing to the presence of calcite in the sample, have been detected. Besides, sulphates were observed through small peaks at 3520 cm^−1^, 3400 cm^−1^, and 1100 cm^−1^, probably associated with small amounts of gypsum present in the mortar samples. Lastly, a small peak of quartz was observed at 1079 cm^−1^. The Roman mortar samples (Rom-02 and Rom-03) present a similar composition: calcite (1404 cm^−1^, 873 cm^−1^, and 712 cm^−1^) and quartz (1079 cm^−1^).

Hematite pigment was identified in the painting layer of the red Arabic samples (Is-06 and Is-08), through the intense peaks observed at 1030 cm^−1^, 540 cm^−1^, and 470 cm^−1^ and a broad band at 3140 cm^−1^, and also, the presence of 3690 cm^−1^ and 1000 cm^−1^ peaks with a lower intensity allows to deduce that a small quantity of red earth was also present in the painting samples. Regarding the white painting layer of the Arabic samples (Is-02 and Is-03), carbonates were detected, where intense peaks were observed at 1404 cm^−1^, 873 cm^−1^, and 712 cm^−1^. On the other hand, in the red painting layer of the Roman sample (Rom-02), red earth was identified (3690 cm^−1^ and 1000 cm^−1^), whereas carbonates (1404 cm^−1^, 873 cm^−1^, and 712 cm^−1^) were detected in the white painting layer of the Roman sample (Rom-03) [16, 17].

## 4. Conclusions

The lack of knowledge about the mural paintings of the archaeological site of Cercadilla has allowed to conduct this work in order to shed light on the materials and techniques used.

In general, the Roman mural painting stratigraphies presented a larger number of overlapped layers (3-4) with a careful selection of raw materials, according to the references consulted. The Arabic sample stratigraphy presents a smaller number of preparatory layers (1-2), and the granulometry of the sand added is thinner. In addition, it was observed that the Arabic paints presented two pictorial layers, probably due to a repaint at some point in the past.

The use of a wide number of analytical techniques showed that the preparatory layers, in Roman and Arabic paints, were mainly composed of calcite and in a lower amount of quartz sand. These were possibly added during its manufacturing, or they could also consist of impurities in the raw material. In the case of the Roman samples, the presence of iron, silicon, aluminium, and potassium (silicate minerals) was also observed. This could be attributed to the use of ceramic fragments in the mortar manufacturing in order to confer hydraulic properties (i.e., *opus caementicium* and *opus signinum* constructive techniques) for the building of the baths.

Regarding the pictorial layers, it is concluded that red earth was used in the Roman paintings as red pigment, whereas calcite was used as white pigment. In the case of the Arabic paints however, ochre pigment was employed. This pigment contains hematite in high concentration and red earth in low proportion. The white pigment in the Arabic samples was mainly composed of calcium carbonate (calcite).

In conclusion, differences in production technology were identified. The Roman samples used a higher quality manufacturing technology than Arabic samples. The use of these kinds of materials, in both periods, concurs in comparison not only with other wall paintings analysed in the Peninsula regardless of whether it was close chronologically or geographically. The use of these materials was due to the proximity of the archaeological enclaves and the abundance of those materials in the area, as much as the same tradition in the elaboration of the mural paintings, which was generally based on Vitruvius treatises. In addition, the use of some materials or others in turn depends on the importance and use of the building.

## Figures and Tables

**Table 1:** Sample description and analysis carried out on each sample.

	Sample code	Colour	Analysis
Roman period (III-IV AD)	Rom-01	Red	OM, SEM-EDX, and WD-XRF
	Rom-02	Red	OM, SEM-EDX, and FT-IR
	Rom-03	White	OM, SEM-EDX, and FT-IR
	Rom-04	Red	OM
	Rom-05	Red	*μ*-XRD
	Rom-06	White	OM, SEM-EDX
	Rom-07	White	OM
	Rom-08	Mortar	XRD
	Rom-09	White	*μ*-XRD
	Rom-10	Mortar	XRD
	Rom-11	Red	OM

Arabic period (X-XI AD)	Is-01	White	OM
	Is-02	White	OM, SEM-EDX, and FT-IR
	Is-03	White	OM, SEM-EDX, *μ*-XRD, and FT-IR
	Is-04	White	OM
	Is-05	Red	*μ*-XRD
	Is-06	Red	OM, SEM-EDX, and FT-IR
	Is-07	Red	OM
	Is-08	Red	OM, SEM-EDX, WD-XRF, XRD, and FT-IR

**Table 2:** Chemical composition of preparatory and pictorial layers from Roman (Rom) and Arabic (Is) samples by SEM-EDX (wt%).

Sample code	Layer	Sublayer	CaO	SiO_2_	Al_2_O_3_	Fe_2_O_3_	K_2_O
Rom-01	Preparation	C1	40,5	59,5	—	—	—
		C2	62,6	37,4	—	—	—
		C3	95,6	4,4	—	—	—
	Pictorial layer	Red	50,1	37,5	8,2	4,2	

Rom-02	Preparation	C1	39,8	50,1	5,3	3,9	0,9
		C2	56,4	31,7	6,5	4,1	1,3
		C3	89,4	10,6	—	—	—
	Pictorial layer	Red	49,3	36,4	9,0	5,3	—

Rom-03	Preparation	C1	33,4	66,6	—	—	—
		C2	33,2	49,9	8,1	—	1,6
		C3	46,6	41,9	7,2	4,3	—
	Pictorial layer	White	86,0	14,0			

Is-02	Preparation	C1	77,5	19,3	1,8	1,3	—
		C2	75,9	20,7	2,1	1,3	—
	Pictorial layer	White	73,0	25,3	0,8	0,8	

Is-03	Preparation	C1	61,0	34,2	3,2	1,6	—
		C2	63,6	33,5	2,1	0,8	—
	Pictorial layer	White	78,5	15,6	3,4	2,4	—

Is-06	Preparation	C1	78,5	21,5	—	—	—
		C2	76,6	23,4	—	—	—
	Pictorial layer	Red	40,0	22,5	4,4	33,2	—

Is-08	Preparation	C1	81,6	14,9	2,5	1,0	—
		C2	71,5	24,0	2,7	1,4	—
	Pictorial layer	Red	39,0	3,3	1,0	56,7	—

Note: C1, C2, and C3 correspond to preparatory layers, from the inner zone to the top, where the pictorial layer is placed. The pictorial layer is described by its colour (red or white).

**Table 3:** Mineralogical composition by X-ray Diffraction for the Roman and Arabic mortar samples.

XRD	Roman sample (Rom-10)	Arabic sample (Is-08)
Calcite	58,1%	95,6%
Quartz	32,0%	2,6%
Phyllosilicate	9,9%	N.D.
Albite	N.D.	0,4%
Gypsum	N.D.	1,4%

N.D.: not detected.

**Table 4:** Mineralogical composition by micro X-ray Diffraction for the Roman and Arabic painting surfaces.

*μ*-XRD	Roman samples		Arabic samples	
	Rom-09 (white)	Rom-05 (red)	Is-03 (white)	Is-05 (red)
Calcite	52,4%	60,3%	57,9%	55,8%
Quartz	11,8%	3,6%	3,3%	12,1%
Gypsum	35,8%	36,1%	38,8%	20,9%
Hematite	N.D.	N.D.	N.D.	11,2%

N.D.: not detected.

**Table 5:** Chemical composition of mortar samples from Roman (Rom) and Arabic (Is) samples by WD-XRF (wt%).

Sample	SiO_2_	Al_2_O_3_	Fe_2_O_3_	MnO	MgO	CaO	Na_2_O	K_2_O	TiO_2_	P_2_O_5_	SO_3_
Rom-01	36,42	8,45	3,40	0,11	1,13	7,97	0,21	2,12	0,34	0,31	0,05
Is-08	12,61	2,33	0,75	0,02	0,59	66,33	0,11	0,50	0,08	0,32	0,28

## Data Availability

All data generated or analysed during this study are included in this article.
